# Maternal Condition but Not Corticosterone Is Linked to Offspring Sex Ratio in a Passerine Bird

**DOI:** 10.1371/journal.pone.0110858

**Published:** 2014-10-27

**Authors:** Lindsay J. Henderson, Neil P. Evans, Britt J. Heidinger, Aileen Adams, Kathryn E. Arnold

**Affiliations:** 1 Department of Neurobiology, Physiology and Behavior, University of California Davis, Davis, California, United States of America; 2 College of Medical, Veterinary & Life Sciences, The University of Glasgow, Glasgow, United Kingdom; 3 Department of Biological Sciences, North Dakota State University, Fargo, North Dakota, United States of America; 4 Environment Department, The University of York, York, United Kingdom; University of Lausanne, Switzerland

## Abstract

There is evidence of offspring sex ratio adjustment in a range of species, but the potential mechanisms remain largely unknown. Elevated maternal corticosterone (CORT) is associated with factors that can favour brood sex ratio adjustment, such as reduced maternal condition, food availability and partner attractiveness. Therefore, the steroid hormone has been suggested to play a key role in sex ratio manipulation. However, despite correlative and causal evidence CORT is linked to sex ratio manipulation in some avian species, the timing of adjustment varies between studies. Consequently, whether CORT is consistently involved in sex-ratio adjustment, and how the hormone acts as a mechanism for this adjustment remains unclear. Here we measured maternal baseline CORT and body condition in free-living blue tits (*Cyanistes caeruleus*) over three years and related these factors to brood sex ratio and nestling quality. In addition, a non-invasive technique was employed to experimentally elevate maternal CORT during egg laying, and its effects upon sex ratio and nestling quality were measured. We found that maternal CORT was not correlated with brood sex ratio, but mothers with elevated CORT fledged lighter offspring. Also, experimental elevation of maternal CORT did not influence brood sex ratio or nestling quality. In one year, mothers in superior body condition produced male biased broods, and maternal condition was positively correlated with both nestling mass and growth rate in all years. Unlike previous studies maternal condition was not correlated with maternal CORT. This study provides evidence that maternal condition is linked to brood sex ratio manipulation in blue tits. However, maternal baseline CORT may not be the mechanistic link between the maternal condition and sex ratio adjustment. Overall, this study serves to highlight the complexity of sex ratio adjustment in birds and the difficulties associated with identifying sex biasing mechanisms.

## Introduction

Maternal quality and natal conditions can affect the survival and reproductive potential of offspring in a sex-specific manner [1], [2]. Therefore mothers breeding in favourable conditions are predicted to gain fitness benefits from investing in the sex that will benefit most from those conditions, and vice versa [2], [3]. In agreement with sex allocation theory there are both experimental and correlative studies that demonstrate that brood sex ratio adjustment is associated maternal condition and natal conditions in avian species [4], [5], [6], [7], [8]. However, the replication of results has proved difficult, with outcomes differing between years and studies [7], [9]. Moreover, predicting the direction of a sex ratio bias has been problematic, with evidence of no bias or the opposing bias from that expected in empirical studies [10], [11], [12]. Overall, the variety of avian life histories, extended parental care and the array of factors that could influence the benefits of sex ratio manipulation cause the prediction of sex ratio adjustment in birds to be complex [13]. In this case, identifying a mechanism of sex ratio adjustment would offer insight into the potential costs of manipulation and may improve predictions of when sex ratio adjustment is expected to occur [14].

In birds females are the heterogametic sex (producing Z- and W-bearing ova), therefore it has been suggested that primary sex ratio adjustment (occurring prior to laying) could be under maternal control [15]. The steroid hormone corticosterone (CORT) has been proposed to play a role in brood sex ratio adjustment, as it is modulated in response to factors implicated in sex ratio adjustment, including maternal body condition, environmental conditions and partner quality [5], [8], [16], [17], [18]. Baseline CORT concentrations fluctuate within individuals in response to the prevalent conditions, to maintain homeostasis and energy-balance through their effects on behaviour and physiological processes [19]. Due to this, elevated baseline CORT has been associated with poor body condition [5], [17], [20], [21], inclement environmental conditions and incompatible breeding partners in birds [22], [23], [24], [25]. Therefore it would be expected to be associated with investment in the sex whose survival and reproductive success is least affected by poor developmental conditions [14], [17]. In agreement with this hypothesis correlative and experimental studies have found a link between elevated CORT and female biased brood sex ratios, in species where males are the larger sex and therefore may be more sensitive to poor natal conditions than females [5], [16], [17]. In addition, in the Gouldian finch (*Erythrura gouldiae*) genetically incompatible breeding pairs have an 84% greater mortality of daughters compared to compatible pairs. Elevated CORT in females constrained to mate with genetically incompatible partners, has been correlatively and causally linked to the adaptive overproduction of male offspring [18], [26].

The mechanism by which CORT could influence the sex of offspring is currently unknown, but could potentially act at the pre- or post-laying stage. A number of hypothesis have been posited including, differential segregation of the sex chromosomes during meiosis [27] or selective reabsorption of ova dependent upon sex, which could result in laying gaps or infertile eggs due to selective fertilization of ova based on sex [14]. Alternatively, yolk CORT concentrations can influence hatching success [28], nestling growth [29] and nestling survival [17], [30], thus could affect brood sex ratio through early embryo death or sex-specific nestling mortality. Finally, mothers with elevated CORT may provide inferior parental care, thus negatively impacting offspring quality and survival [17]. The methods that have been used to experimentally elevate CORT and investigate the effects upon sex ratio do not provide unequivocal evidence to establish the mechanism through which CORT acts. For example, CORT has been experimentally elevated within baseline and above baseline levels for prolonged periods (days) during egg laying [16], [17], [26]. This method could have knock-on physiological effects, such as changes in glucose availability, which has also been implicated in brood sex ratio adjustment [31]. This methodology could also influence maternal care and yolk concentrations [17], which could result in sex ratio adjustment after laying. Interestingly, studies provide evidence for both of these hypotheses as there is evidence of causal relationship between maternal baseline CORT and brood sex ratio both at laying [5], [11], [16] and at fledging [17]. Alternatively, to address whether CORT directly affects offspring sex, other studies have elevated CORT transiently during sex determining meiosis [11], [12]. However, these studies have used pharmacological CORT concentrations and have resulted in a offspring sex ratio bias opposite to that predicted from sex allocation theory [11], [12]. Ultimately, there remains a lack of consensus as to how maternal CORT may cause brood sex ratio adjustment.

There have been a number of studies that present evidence of brood sex ratio adjustment in the blue tit (*Cyanistes caeruleus*) [32], [33], [34], [35], [36]. Specifically, studies have examined whether females paired with attractive males, can increase their fitness by investing in sons rather than daughters, as sons may inherit their fathers attractiveness [34]. Although there is some correlative and causal evidence of a link between paternal attractiveness and male-biased brood sex ratio in blue tits, the results have proved difficult to replicate and have varied between years [33], [36]. As maternal baseline CORT has been shown to be higher in females with unattractive mates [37], [38], CORT could be the mechanism through which females could adjust brood sex ratio in response to male attractiveness [18]. There is also evidence for assortative mating in blue tits [39], thus females paired with high quality attractive partners may also be of superior quality. However, to date the relationship between maternal condition, maternal baseline CORT and brood sex ratio has not been investigated in blue tits.

In this study, a free-living population of blue tits were monitored for three years to assess the relationship between maternal baseline CORT, maternal condition and brood sex ratio. To identify the potential mechanisms of sex ratio adjustment, both primary and secondary sex ratio were established. In addition, laying gaps were recorded to identify evidence of potential reabsorption of ova, and un-hatched eggs were analysed to test for sex-biased fertilization or embryo death. Nestling mass and growth were also measured to investigate whether maternal baseline CORT and/or maternal condition had sex-specific effects on nestlings, therefore indicating the potential benefits of brood sex ratio adjustment. In the final year of the study a field-based experiment was conducted to investigate whether transient elevation of maternal CORT at sex determining meiosis during egg laying influenced brood sex ratio. In order to ensure CORT concentrations were elevated during sex determination we acutely elevated CORT above baseline levels but within the physiological range for this species. We predicted that reduced maternal condition and elevated maternal CORT would be associated with reduced nestling quality and a female biased sex ratio, as males are the larger sex and may be more sensitive to poor conditions.

The main aims of this study were to investigate whether; 1) endogenous maternal baseline CORT and maternal body condition were correlated with primary or secondary brood sex ratio, 2) maternal condition and/or baseline CORT concentrations were related to nestling mass and growth rate, 3) maternal baseline CORT and body condition were correlated and 4) experimental elevation of maternal CORT during egg laying influenced brood sex ratio and offspring quality.

## Methods

### Empirical study

Blue tits breeding in nest boxes in oak-dominated woodland around Loch Lomond, Scotland (56° 13′ N, 4° 13′ W) were studied for three years from April to June 2008–2010. Nest boxes were monitored regularly from the onset of nest building to establish the date the first egg was laid (lay date). Nests were then checked every second day and eggs were counted to establish clutch size. In addition, as blue tits lay one egg per day [40], this allowed laying gaps to be identified. When eggs were found to be warm and no new eggs had been laid on 2 consecutive visits, incubation was deemed to have started and mothers were left undisturbed for 10 days. Nests were then visited every day to establish hatching date, when>50% of eggs had hatched this was considered day 1. All un-hatched eggs and dead nestlings were collected for molecular sexing (see Molecular sex identification).

To measure maternal baseline CORT, birds were caught during provisioning, on day 5–7 after hatching. Mothers were captured on the nest by blocking the entrance hole, and a small blood sample was obtained (about 80–100 µl) after puncture of the brachial vein. All samples were collected within 3 minutes of initial blockage of the nest box entrance. Blood samples were immediately stored on ice and separated through centrifugation within 2 h of collection. Occasionally researchers had to wait for a short period near the nest for birds to enter the nest box, when this occurred the duration of the time at the nest was noted. CORT samples were considered to be baseline because time spent at the nest before capture, time between sampling and blockage of the nest box entrance and time of day were not related to maternal CORT (GLM: *n* = 79, time at nest; *t* = 1.26, *P* = 0.21, sampling time; *t* = −0.01, *P* = 0.99 and time of day; *t* = −1.25, *P* = 0.22). The plasma portion of the sample was removed and stored at −20°C until assay. Circulating CORT concentrations were measured using a double antibody radioimmunoassay (for full details see [41]). CORT was measured in three assays for which the detection limit was 0.03 ng/ml and the intra-and inter-assay variation was 9±2% and 10±5% respectively.

**Figure 1:**
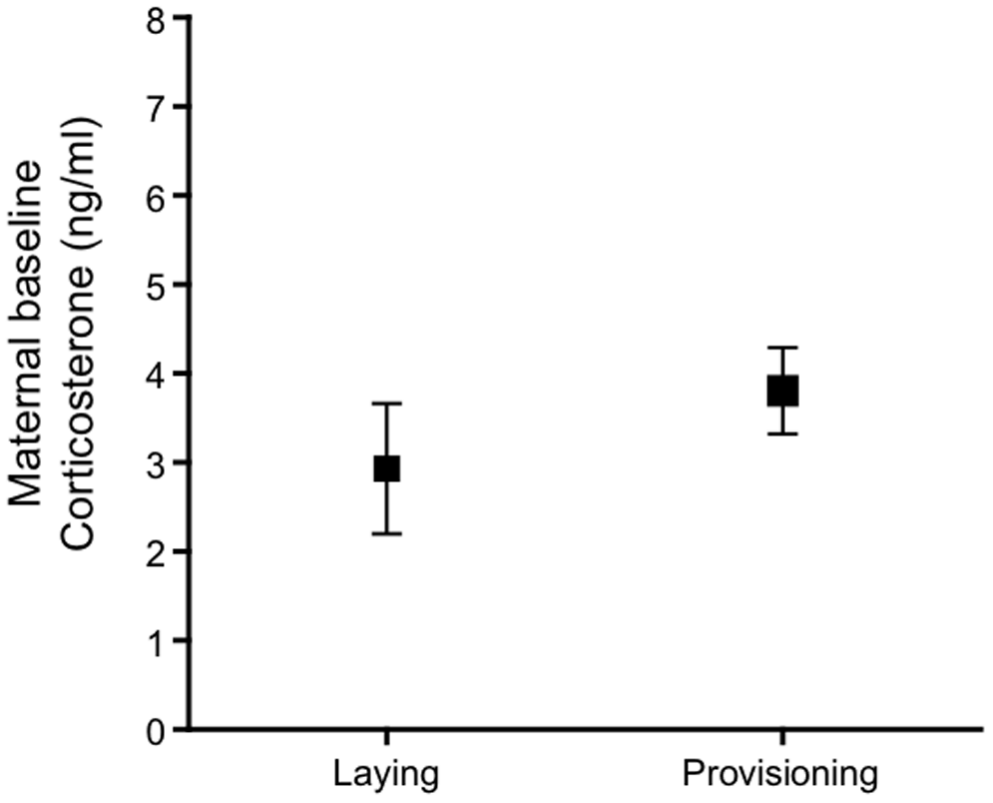
Maternal baseline CORT did not significantly differ when measured during egg laying versus nestling provisioning (*n* = laying: 9, provisioning: 10). Graph shows mean±SE.

Baseline CORT was measured during provisioning rather than egg-laying for two reasons, i) previous studies that have found a link between maternal baseline CORT and brood sex ratio, have blood sampled mothers post egg-laying [5], [16], and ii) baseline CORT concentrations did not differ significantly between females measured during laying or provisioning (figure 1, females matched based on lay date, *n* = laying: 9, provisioning: 10, *t*-Test: *t* = −1.08, *P* = 0.30).

To establish body condition, mothers were weighed to the nearest 0.05 g with a Pesola spring balance and wing length was measured. Maternal condition was established by taking the residuals from a linear regression of mass (g) and wing length (mm). Parental birds were sexed based on presence/absence of a brood patch and aged based on plumage characteristics [42] and all birds captured were fitted with a uniquely numbered aluminium ring (British Trust for Ornithology). As some mothers were measured for body condition but not baseline CORT sample sizes differ between analyses.

### Experimental manipulation of maternal CORT

To deliver CORT non-invasively to breeding blue tits, CORT solution was injected into mealworms that were placed in nest boxes during egg-laying. Sex determining meiotic division occurs 2–4 hrs before ovulation [43], with oviposition occurring approximately 24 hrs later [44], [45]. As blue tits lay in the early hours of the morning [46], sex determination is expected to occur during the night, approximately 28 hrs before oviposition. Therefore, mothers were fed mealworms injected with exogenous CORT or without (Control) in the evening to coincide with sex determination to establish whether maternal CORT influenced the sex ratio at laying. We used a transient method of CORT elevation to control for knock-on physiological effects that could result from chronic elevation of CORT.

Crystalline CORT (Sigma) was dissolved directly into peanut oil (Sigma) through sonification. To ensure the CORT was dissolved evenly in the peanut oil, the solution was sonicated before each use. Mealworms were injected with 20µl of peanut oil containing one of the following concentrations of CORT: (1) Control, no CORT or (2) CORT, 0.3 mg/ml. Hence, mothers received 0 or 6µg CORT per mealworm. We injected solution into mealworms with a 25-μl Hamilton syringe using a 26-gauge ½-inch needle. Prior to injection, mealworms were kept at −20°C to reduce movement during injection. The needle was inserted ventrally, into the anterior abdomen, between two segments. If fluid leaked from the mealworm after injection, it was not used. We used mealworms of approximately 20 mm and 0.1 g in size. This methodology was validated under lab conditions where blue tits fed 6µg CORT injected mealworms had significantly higher CORT 10 minutes after ingestion than blue tits fed Control mealworms (CORT: *n* = 4, mean±SE, 39.9±4.0 ng/ml, Control: *n* = 4, 3.1±0.95 ng/ml, *t*-Test: *t* = 5.54, *P* = 0.001). CORT concentrations did not differ significantly between groups 30 mins after ingestion (*t*-Test: *t* = 0.96, *P* = 0.41). The circulating CORT concentrations achieved after spiked mealworm consumption were within 1 SE of the mean concentrations (*n* = 5, mean±SE, 28.2±11.2 ng/ml) found in blue tits after subjection to a standardized capture and restraint procedure [47]. Therefore, CORT concentrations were elevated within the natural range for this species. For full validation methods please see Materials S1.

In 2010 prior to the onset of nest building, small plastic cups (40×20 mm) were secured on the inside of all nest boxes to later provide a tray for mealworms. Nest boxes were monitored weekly to establish the onset of nest building. When nests were found to be half to fully constructed they were checked daily for eggs. When the first egg was laid, the nest was randomly assigned by the toss of a coin to the CORT or Control group. Beginning that day, a CORT spiked (6 µg) or Control mealworm was placed into the plastic tray every evening between 17:30 and 19:30, until no more eggs had been laid on 2 consecutive visits. This time was chosen because female blue tits have been found to roost as early as 19:00 during egg laying in Scotland [48]. Mothers received their first mealworm to coincide with the sex determination of their third egg. The mean clutch size was 10.9±1.5, consequently on average>80% of eggs laid were manipulated. If we assume a 0.5 sex ratio for the two un-manipulated eggs, with a clutch size of 10, a consistent female sex bias caused by our experiment of>0.1 in the manipulated eggs, would cause a brood sex ratio of <0.42.

To check that the treatment targeted female rather than male blue tits, a hide was erected close to a sub-sample of nest boxes during egg laying (*n* = CORT: 4, Control: 3). The nests were then monitored after the mealworm was placed in the nest until sunset and then checked the following morning before 06:00. This was to record activity at the nest during this time and to establish if the mealworm had been consumed during the night. The mealworm was consumed by 06:00 for each nest box checked. On only one occasion a bird was recorded to enter the nest on more than one occasion before roosting. For all other nests only one bird was recorded to enter the nest and not leave. As female rather than male breeding blue tits roost in the nest box during laying [48], when only one bird was seen entering and not leaving the nest before sunset, it was assumed to be the female. Therefore our observations suggest that only mothers consumed the mealworms between 19:00–06:00. The progress of all manipulated nests were followed as previously described, and nestlings weighed and sexed as below.

### Nestling condition

All individually marked nestlings were weighed to the nearest 0.01 g with a digital balance on day 4, 6, 8, 10 and 14 after hatching. Nestling growth rate was calculated individually as mass gain day^−1^ from day 4–14 after hatching. On day 14 chicks were fitted with a uniquely numbered aluminium ring (British Trust for Ornithology).

### Molecular sex identification

At the age of 14 days, nestlings were blood sampled to provide DNA for molecular sex identification. All nestlings that were collected dead before this time and un-hatched eggs where development was evident were also sexed. A salt extraction [49] or Qiagen DNeasy kits were employed for DNA extraction. Primers were P2/P8 [50]. PCR amplification was carried out in a total volume of 10µl. The final reaction conditions were as follows: 0.8µM of each primer, 200µM of each dNTP, target DNA, 0.35 units GoTaq polymerase (Promega), 2µM (5×) GoTaq Flexi Buffer (Promega) and 2µM of 25 mM MgCl_2_. Thermal cycling was carried out in a Biometra UnoII: 94°C/2 min, 30 cycles of (49°C/40 s, 72°C/40 s and 94°C/30 s), 49°C/1 min, 72°C/5 min. PCR products were separated by electrophoresis on a 2% agarose gel stained with ethidium bromide.

Where possible both the primary sex ratio (Pri), i.e. the sex ratio of all eggs laid, and the secondary sex ratio (Sec), i.e. the sex ratio of all nestlings that fledged was calculated for each nest. However, some of the un-hatched eggs collected showed no evidence of development, therefore they could not be sexed and are hereon referred to as ‘unviable’ eggs. In this case, the nests were included in primary sex ratio analysis if all the remaining offspring were sexed. For some nests it was not possible to calculate primary sex ratio as dead nestlings or eggs were lost prior to sexing, usually because the mother removed them from the nest. In this case only secondary sex ratio was calculated. Due to this samples sizes of primary and secondary sex ratio differ. Overall 91.6% of eggs laid were sexed (*n* = 1511) from 150 un-manipulated broods. Nests were not included in the analysis if any eggs were accidentally broken or molecular sexing was not successful for an individual egg or chick.

### Ethical note

In order to minimise disruption to parents and nestlings a number of precautions were followed. All adult birds were captured and blood sampled within 15 minutes of initial disturbance at the nest, on the majority of occasions birds were disturbed for less than 10 minutes. Furthermore, nestlings were held on heat pads to prevent them from getting chilled and never disturbed for longer than 30 minutes. During the experimental study, nest visits were carried out as quickly as possible to minimise disturbance, and because maternal CORT was elevated within the natural range recorded for this species, the physiological stress mothers were subjected to was comparable to concentrations commonly experienced by the birds. All work was conducted under licence from the UK Home Office and was subject to review by the University of Glasgow ethics committee. Fieldwork was conducted on land leased by The University of Glasgow. This study did not involve endangered or protected species.

### Statistical analysis

Generalized Linear Models (GLMs) with binomial errors and no explanatory terms were used to test if brood sex ratios differed from a binomial distribution [36], [51]. To investigate whether year, maternal body condition, maternal baseline CORT, lay date, and maternal age explained variation in brood sex ratio, GLMs with a binomial error structure and a logit link function, weighted by brood size were used [51]. Overdispersion was not deemed a problem as the residual mean deviance (residual deviance/residual d.*f.*) was always less than 1.5 [51]. All CORT data were square root transformed because of non-normality. GLMs were used to establish whether maternal condition, lay date and age explained variance in maternal baseline CORT. GLMs with a binomial error structure were employed to establish whether the occurrence of laying gaps and unviable eggs (present:1, absent:0) were related to year, experimental treatment, maternal baseline CORT and maternal body condition. General Linear Mixed Models (GLMMs), with brood ID as the random factor, were employed to assess whether nestling mass on day 14 after hatching and nestling growth rate were explained by year, sex, lay date, brood size, maternal baseline CORT and maternal condition. All models were run twice, once with maternal CORT and once with maternal condition. In addition, all two-way interactions with year or sex, and the interaction between maternal body condition and baseline CORT were included in full models.

For the experimental study in 2010, GLMs were used to establish whether maternal condition, maternal CORT and lay date differed between control, CORT and un-manipulated broods. GLMs with a Poisson error structure were used to compare clutch size and number fledged between treatment groups. Similar to the empirical study, Generalized Linear Models with a binomial error structure and a logit link function, weighted by brood size were used to investigate whether the treatment group affected brood sex ratio [51]. GLMMs were used to establish whether nestling mass on day 14 after hatching and nestling growth rate were affected by the treatment.

All models were optimised using backward elimination of non-significant terms. Model validations were applied where appropriate and the underlying statistical assumptions of normality and homogeneity of variance were verified. All statistical analyses were conducted using R version 2.8.0, and the nlme library was used for GLMMs. For raw data please see Data S1.

## Results

### Population brood sex ratio

Population primary and secondary brood sex ratio did not depart from a binomial distribution (Pri: *z* = 1.37, *P* = 0.17, Sec: *z* = 0.68, *P* = 0.49). Furthermore, population brood sex ratios did not differ between years (Pri: *z* = −0.60, *P* = 0.55, Sec: *z* = −1.40, *P* = 0.16).

When the sex of both un-hatched eggs and nestlings that died before fledging was analysed, there was no indication of sex-biased offspring mortality (Yates' corrected: *n* = M: 37, F: 30, X^2^ = 0.73, *P* = 0.39). The number of mothers that exhibited laying gaps did not differ between years (*z* = −0.55, *P* = 0.58). However, the number of mothers that laid unviable eggs (where development was not detected) was significantly lower in 2010 compared with the other two years, with 55%, 38% and 18% of broods containing unviable eggs in 2008–2010 respectively (*z* = −3.21, *P* = 0.001).

### Maternal baseline corticosterone, body condition and brood sex ratio

**Figure 2:**
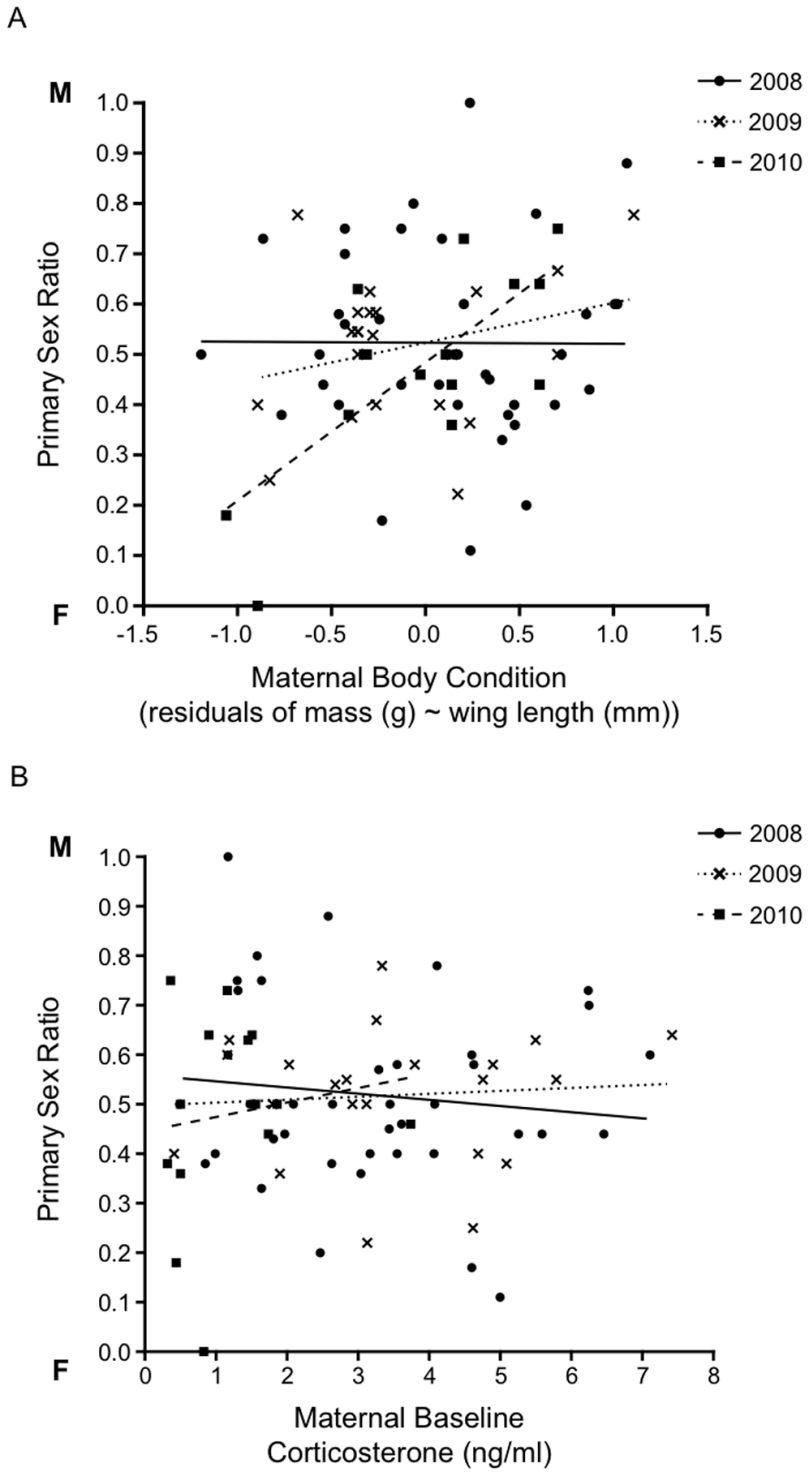
The relationship between primary brood sex ratio and a) maternal body condition (*n* = 89) and b) maternal baseline CORT (*n* = 76) from 2008–2010. M indicates a male and F indicates a female biased brood sex ratio.

Maternal body condition was significantly correlated with both primary and secondary brood sex ratio in a year-specific manner (table 1). In 2010 only, mothers in superior condition had more male biased broods (figure 2a, Pri: *z* = 3.12, *P* = 0.002, Sec: *z* = 3.19, *P* = 0.001). Maternal CORT (figure 2b, Pri: *z* = 0.30, *P* = 0.77, Sec: *z* = 0.51, *P* = 0.61), maternal age and lay date were not related to brood sex ratio.

**Table 1:** The results of GLMs investigating whether maternal body condition influenced a) primary (*n* = 89) and b) secondary (*n* = 88) brood sex ratio.

Factor	d.f. Effect	SE	*z*	*P*
**a) Primary**				
Year	2	0.24	0.21	0.84
Maternal condition	1	0.19	−0.17	0.86
Age	1	0.17	−0.04	0.97
Lay date	1	0.02	0.64	0.52
Maternal condition *Year	2	0.38	2.75	**0.006**
**b) Secondary**				
Year	2	0.25	−0.40	0.69
Maternal condition	1	0.20	−0.16	0.87
Age	1	0.16	−0.05	0.96
Lay date	1	0.03	0.55	0.58
Maternal condition *Year	2	0.37	2.81	**0.005**

Maternal body condition and CORT did not predict whether mothers exhibited laying gaps (CORT: *z* = −0.99, *P* = 0.32, Condition: *z* = 0.38, *P* = 0.70). Also, whether mothers laid unviable eggs was not associated with maternal body condition or CORT (CORT: *z* = −0.09, *P* = 0.93, Condition: *z* = 0.17, *P* = 0.86).

### Maternal baseline corticosterone, body condition and nestling condition

**Figure 3:**
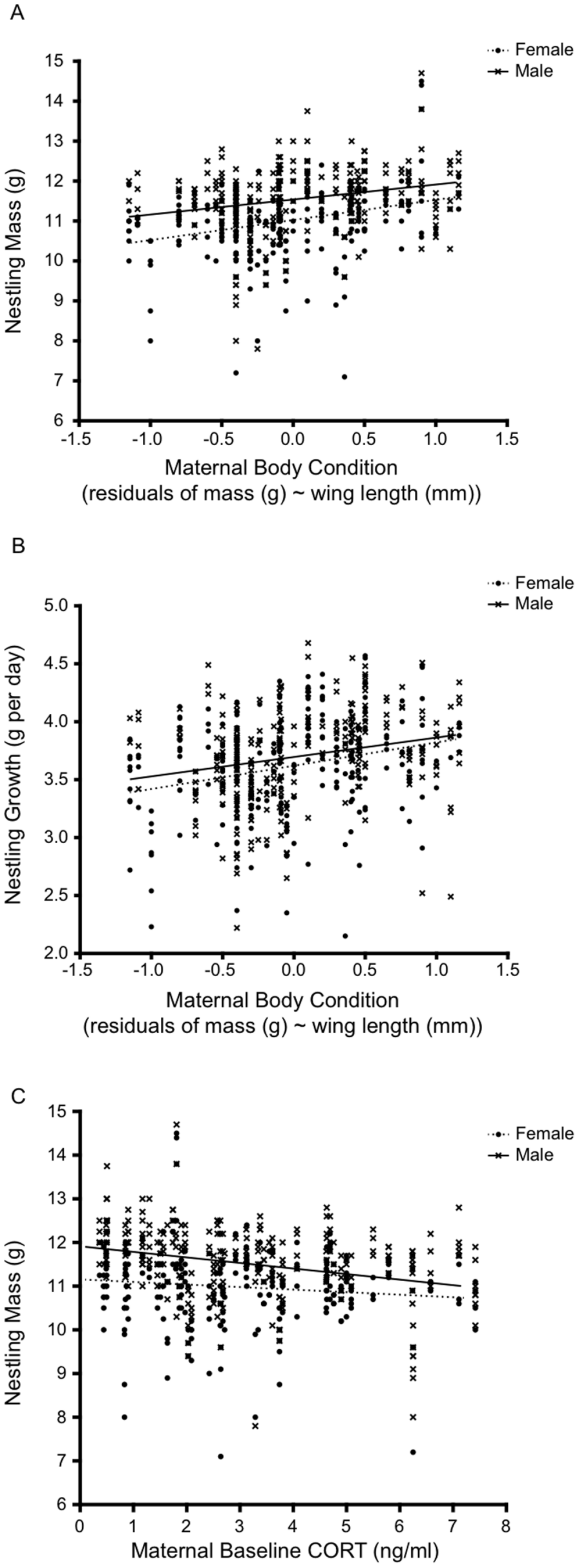
The relationship between a) maternal body condition and nestling mass on day 14 after hatching, b) maternal body condition and nestling growth rate, and c) maternal baseline CORT and nestling mass on day 14 after hatching from 2008–2010 (*n* = chicks: 520, broods: 57).

In all years, mothers in superior body condition had heavier and faster growing offspring than mothers in poor condition (table 2, figure 3ab). Maternal body condition did not influence nestling mass or growth in a sex-specific manner (Mass: Body Condition × Sex; *t* = −1.00, *P* = 0.32 and Growth: Body Condition × Sex: *t* = −0.65, *P* = 0.52). Male nestlings were consistently heavier and grew at a faster rate than their female siblings in all years (table 2, figure 3ab). Nestling mass and growth rate did not differ between the years (table 2).

**Table 2:** The results of GLMs investigating whether maternal body condition influenced a) nestling mass and b) nestling growth (broods = 59).

Factor	d.f. Effect	SE	*t*	*P*
**a) Nestling mass**				
Year	2	0.23	1.11	0.27
Sex	1	0.05	10.97	**<0.001**
Maternal condition	1	0.17	3.09	**0.003**
**b) Nestling growth**				
Year	2	0.10	−0.51	0.61
Sex	1	0.02	5.06	**<0.001**
Brood size	1	0.01	−2.06	**0.04**
Maternal condition	1	0.07	2.37	**0.02**

Maternal baseline CORT was negatively correlated with nestling mass, but not nestling growth (figure 3c, Mass; *t* = −2.18, *P* = 0.03 and Growth; *t* = −1.60, *P* = 0.12). Maternal baseline CORT did not explain variation in nestling mass or growth in a sex-specific manner (Mass: Sex × CORT; *t* = −0.90, *P* = 0.37, Growth: Sex × CORT; *t* = −1.59, *P* = 0.11).

### Maternal baseline corticosterone and body condition

**Figure 4:**
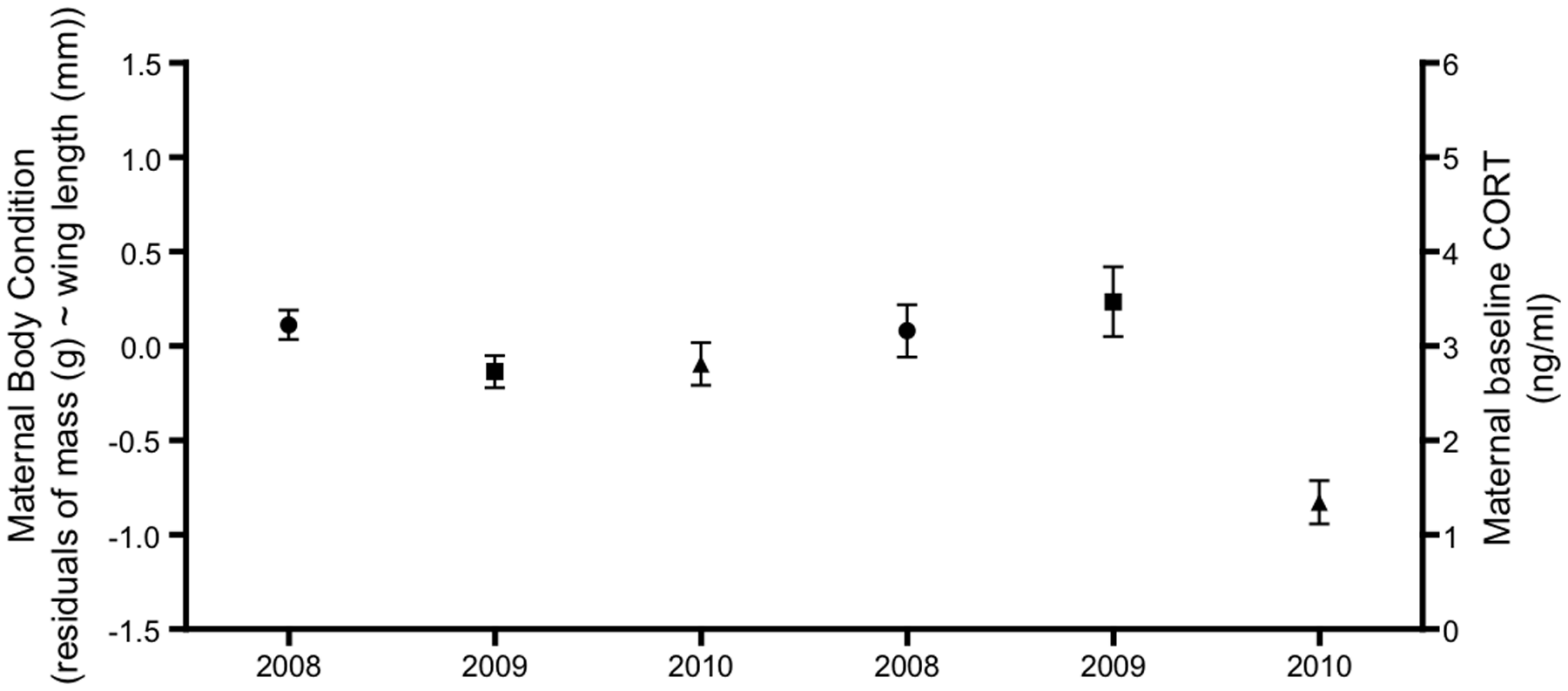
Inter-annual variation in maternal baseline CORT (*n* = 76) and maternal body condition (*n* = 89) from 2008–2010.

In 2008 and 2009, maternal CORT was significantly higher than 2010 (figure 4, *t* = −4.39, *P* <0.001). However, maternal body condition was significantly lower in 2009 compared with the other two years (figure 4, *t* = −2.00, *P* = 0.048). Maternal body condition did not explain variation in maternal baseline CORT (*t* = −1.02, *P* = 0.31).

### Experimental study

**Figure 5:**
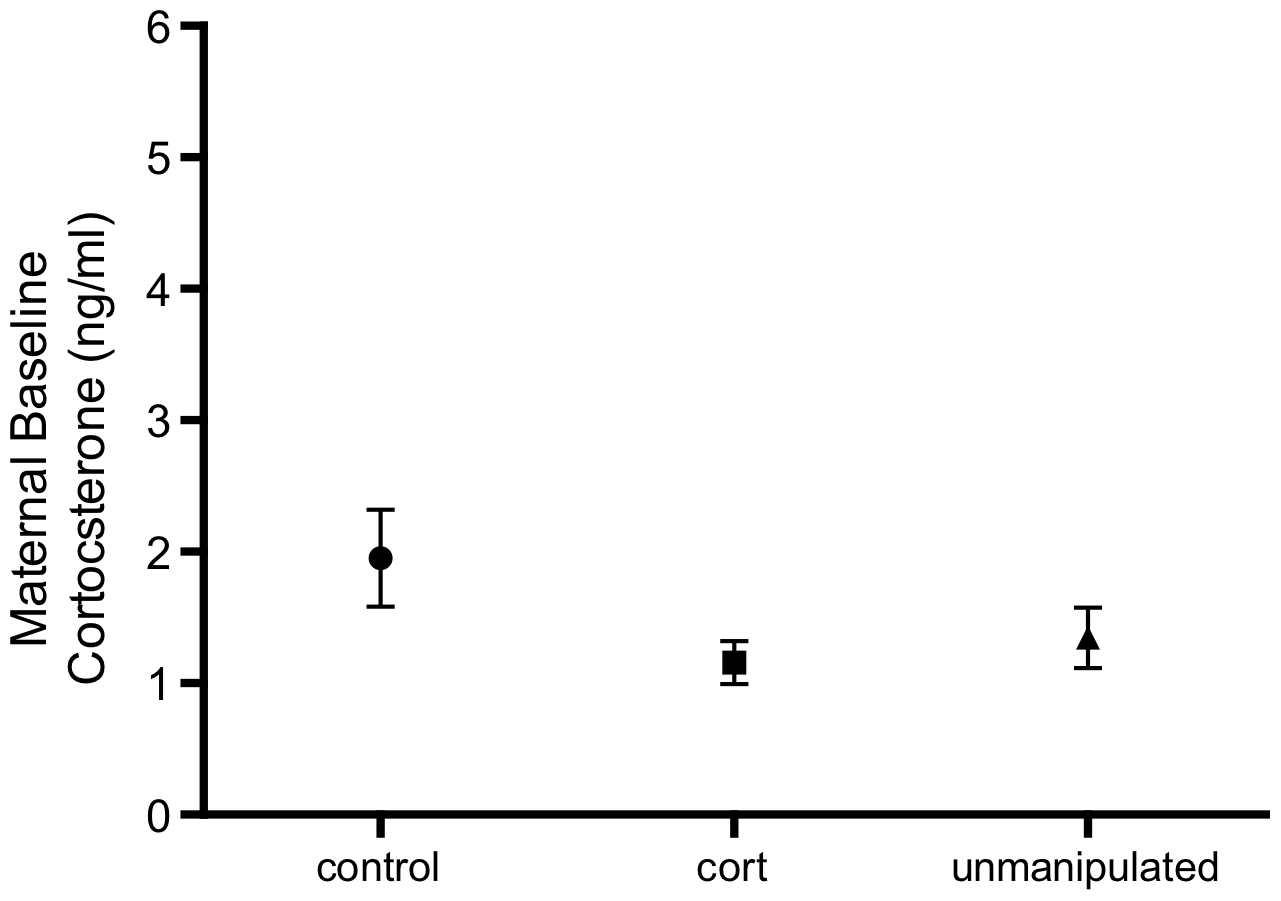
Maternal baseline CORT between experimental treatments. CORT: mothers fed CORT spiked mealworms during egg laying (*n* = 15), Control: mothers fed mealworms (*n* = 13) and un-manipulated mothers (*n* = 17).

There were 48 nests manipulated and monitored until fledging in 2010 (*n* = Control 24, CORT 24). In addition, we matched all experimental nests by date with 24 un-manipulated control nests. Lay date (*t* = 0.33, *P* = 0.74), clutch size (*z* = 0.22, *P* = 0.83), number fledged (*z* = −0.32, *P* = 0.74), maternal body condition (*t* = 0.56, *P* = 0.58) and maternal CORT (figure 5, *t* = −1.76, *P* = 0.09) did not differ between treatments. In addition, the number of mothers that exhibited laying gaps (*z* = −0.56, *P* = 0.58) and laid unviable eggs (*z* = −1.49, *P* = 0.14) did not differ between the treatments.

**Figure 6:**
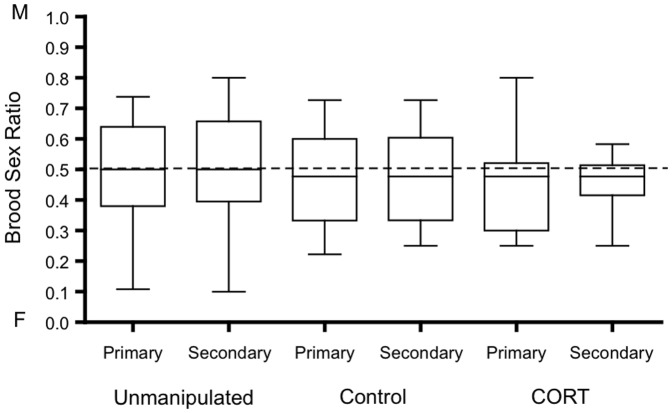
Primary and secondary brood sex ratios between experimental treatments. CORT: mothers fed CORT spiked mealworms during egg laying (*n* = 24), Control: mothers fed mealworms (*n* = 24) or un-manipulated mothers (*n* = 24). Graph shows median and interquartile range. M indicates a male and F indicates a female biased brood sex ratio. Dashed line denotes a 50∶50 brood sex ratio.

Primary and secondary brood sex ratios did not differ between treatments (figure 6, Pri: *z* = −0.47, *P* = 0.64, Sec: *z* = −0.05, *P* = 0.96). There was a marginally non-significant trend that the interaction term Treatment x Maternal body condition predicted offspring sex ratio (Pri: *z* = 1.86, *P* = 0.06, Sec: *z* = 1.80, *P* = 0.07). When the treatments were analysed individually, maternal body condition was positively correlated with male biased broods in the un-manipulated group only (Pri: Un-manipulated, *z* = 3.02, *P* = 0.002, Control, *z* = 0.40, *P* = 0.69, CORT, *z* = −0.07, *P* = 0.95; Sec: Un-manipulated, *z* = 3.08, *P* = 0.002, Control, *z* = 0.56, *P* = 0.56, CORT, *z* = −0.24, *P* = 0.81).

Nestling mass and growth rate did not differ between treatments (Mass: *t* = −0.89, *P* = 0.38 and Growth: *t* = −0.49, *P* = 0.63), and were not affected by treatment in a sex-specific manner (Mass: Sex × Treatment; *t* = 0.002, *P* = 0.99 and Growth: Sex × Treatment; *t* = 0.68, *P* = 0.50). Similar to un-manipulated nests, male nestlings from manipulated broods grew at a significantly faster rate than their female siblings (*t* = 5.12, *P* <0.001) and were heavier (*t* = 8.47, *P* <0.001).

## Discussion

Our results suggest that maternal CORT is not related to brood sex ratio adjustment in the blue tit. Furthermore, unlike previous studies maternal baseline CORT was not correlated with maternal condition [5], [17]. In agreement with this, exogenous elevation of maternal CORT during egg laying did not influence offspring sex ratio. However, mothers in superior condition produced male biased broods in one year of the study, and both maternal body condition and baseline CORT were associated with indices of nestling quality. Specifically, maternal CORT was negatively correlated with nestling mass, but not growth rate, whereas, maternal condition was positively correlated with both nestling mass and growth rate. This result suggests that maternal condition is more closely related to offspring quality than baseline CORT. Although male nestlings were heavier and grew at a faster rate than female nestlings, maternal condition and CORT concentrations did not influence nestling condition in a sex-specific manner.

Studies that have found a link between maternal CORT and brood sex ratio have also found maternal CORT to be associated with factors that may influence the adaptive significance of sex ratio adjustment, i.e. maternal condition [5], [17], environmental conditions [8], [52] and mate attractiveness [18], [38]. In the present study however, maternal condition was linked to brood sex ratio in one year, but was not correlated with maternal CORT in any year. Furthermore, a previous study that found a correlation between maternal CORT and sex ratio manipulation, also found sex-specific effects of elevated maternal CORT upon nestling mass [17]. However, our results do not support sex-specific effects of maternal CORT upon nestling condition. Therefore, in blue tits maternal CORT may not be indicative of circumstances that might favour sex ratio adjustment, and thus may not be expected to influence brood sex ratio.

Our results provide evidence that CORT may not be directly linked to sex ratio adjustment consistently across bird species. Previous studies that present evidence of a link between maternal CORT and offspring sex ratio differ in the timing of sex ratio adjustment, and therefore the potential mechanisms employed. For example, there is evidence of a pre-laying mechanism in peafowl (*Pavo cristatus*), Japanese quail (*Coturnix coturnix japonica*), Gouldian finch and white-crowned sparrows (*Zonotrichia leucophrys*) [5], [16], [18], [26], [38], as maternal baseline CORT was found to be correlated and causally linked to the primary sex ratio in these species. However, in the European starling (*Sturnus vulgaris*), exogenous elevation of maternal CORT during egg laying was associated with secondary brood sex ratio adjustment through male nestling mortality [17]. The lack of a relationship between maternal CORT, brood sex ratio and nestling mortality in this study and the contrasting findings of previous studies highlight the need for additional research to establish the generality of hormonal mechanisms in sex ratio manipulation and the timing of these adjustments.

Importantly, there were limitations of the methods employed in this study. Although previous studies have found a significant relationship between maternal baseline CORT measured post-laying and brood sex ratio [5], [16], it would have been preferable to measure CORT during egg laying to coincide with the sex determination of chicks. Furthermore, although CORT concentrations did not differ between breeding stages in our population, and there is evidence that CORT concentrations are consistent between breeding stages within individuals [53], [54], this could not be ascertained in this study. Therefore, the CORT concentrations measured during brood rearing may not have reflected those experienced by mothers during egg laying. However, it is important to note that the probability of nest desertion is high in wild birds when they are blood sampled during early breeding stages [55]. This is an important limitation for studies of wild birds, especially in species such as the blue tit, where re-nesting is not possible due to the short duration of abundant food that is essential for breeding [40].

The method used in this study to elevate maternal CORT was non-invasive, and elevated CORT concentrations above baseline to stress-induced levels for a transitory period during sex determining meiosis. As mothers began the treatment once they had laid their first egg, only ∼80% of each clutch was manipulated (out of mean brood size of 10). Our results showed that the primary and secondary brood sex ratios produced after the CORT treatment were 0.47 and 0.45 respectively. Therefore the mean brood sex ratio did not deviate>0.1 from parity, when taking into account that the first two eggs were not manipulated. Due to the limitations of our manipulation we would not have been able to identify a change of <0.1 in brood sex ratio. However, an adjustment of <0.1 would be an extremely small change in brood sex ratio that would be unlikely to affect maternal fitness. Also, as the study was field based it was not possible to monitor hatching, therefore we could not establish the laying order of chicks. Previous studies provide evidence that birds can manipulate the sex of offspring adaptively dependent upon the order that they are laid [56]. Thus future studies that take laying order in account would be insightful.

There is evidence of both a correlative and causal link between maternal condition and brood sex ratio from a wide range of avian species [4], [5], [57], [58]. However, there are also studies that have found no such relationship [9], [59], [60], [61]. In addition, studies that have measured maternal condition and offspring sex ratio over multiple years in birds are rare, and where contrasting patterns between years have been found convincing biological explanations are lacking [9], [62]. There is evidence to suggest that the link between maternal condition and offspring sex ratio is influenced by the prevalent conditions. In red deer (*Cervus elaphus*) the tendency of dominant females to produce more male offspring disappeared as population density increased [52], which has been suggested to have been caused by increased mortality of male foetuses as conditions became less favourable [63]. In our study, maternal condition was related to offspring sex ratio in one year of our study (2010). In 2010 there were significantly fewer mothers that laid unviable eggs compared with the two other years. Thus if the unviable eggs in the previous two years were male this may have obscured the effect of maternal condition upon sex ratio. However, the incidence of unviable eggs was not linked to maternal condition, and when the unviable eggs were considered male and the data re-analysed, maternal condition remained non-significantly correlated with brood sex ratio in two out of the three years (maternal condition × year: *z* = 2.72, *P* = 0.01). Therefore it is unlikely that sex-biased early embryo death or fertilization of ova obscured sex ratio adjustment in these years.

Interestingly, the results of our experiment showed that our manipulation negated the correlation between maternal body condition and brood sex ratio in 2010. In both the CORT and control groups there was no correlation between maternal body condition and brood sex ratio, but there was a positive correlation between body condition and brood sex ratio in un-manipulated broods. A recent study has shown that CORT manipulation during laying can influence the relationship between maternal condition and brood sex ratio. For example, exogenous CORT treatment caused a negative relationship between maternal body mass and brood sex ratio in chickens, whereas in the control group there was a positive correlation between body mass and sex ratio [64]. Overall, these results provide evidence of the context-dependence of the relationship between maternal condition and brood sex ratio.

Variation in the breeding conditions between years could influence the fitness benefits of sex ratio adjustment in relation to maternal condition. In the great tit (*Parus major*) a closely related species, natal conditions influence lifetime reproductive success more strongly in male compared with female birds [65]. Therefore, mothers in superior condition may derive fitness benefits from investing in sons only when breeding conditions are good. In our study, the year that brood sex ratio was linked to maternal condition (2010) was characterized by lower maternal CORT and relatively good maternal condition, most likely due to high food availability and favourable weather conditions in this year compared with the other years [66]. However, manipulative studies are required to provide convincing evidence of a link between maternal condition and brood sex ratio adjustment in this species.

Mothers in superior condition had heavier, faster growing offspring, but these effects were not sex specific. Therefore, although male nestlings grew at a faster rate and were heavier than females, maternal condition did not influence growth or mass more strongly in sons compared with daughters. In spite of this, mothers may have improved their fitness by investing in sons when they were in superior condition, as improved nestling mass and growth during the nestling phase can have beneficial long-term effects for male but not female birds. For example in the great tit, improved nestling mass close to fledging was linked to greater reproductive success in sons but not daughters [67]. Unfortunately, it was not possible in our study to investigate the effects of maternal condition upon the future reproductive success of offspring, as few nestlings were re-captured in subsequent years.

Our finding that the relationship between maternal condition and brood sex ratio adjustment differed between years is consistent with previous studies in the blue tit. Evidence in support of a relationship between paternal attractiveness and offspring sex ratio varies both between years and populations [32], [33], [34], [35], [36]. This may suggest that brood sex ratio manipulation is non-adaptive or constrained by chromosomal sex determination, or that the optimal sex ratio varies across years dependent upon the prevalent conditions [36]. However, as previous studies have shown that temporal change in maternal body condition during breeding predicts offspring sex ratio rather than absolute values [68], [69], the lack of relationship in two years of our study may have been because we employed a single measure of maternal body condition.

Ultimately, their large brood size, limited size dimorphism (∼5%) and variation in extra-pair paternity and thus male reproductive success [70], [71], may suggest that there is weak selection on sex ratio adjustment in the blue tit compared with other species. However, to establish whether the contrasting relationships between parental quality and brood sex ratio across years are significant, further knowledge concerning the fitness benefits of sex ratio adjustment in relation to parental quality across multiple years and environmental conditions are required.

### Conclusions

This study does not provide evidence that maternal baseline CORT plays a role in brood sex ratio adjustment in the blue tit. Our results do provide some evidence that maternal condition was linked to offspring sex ratio adjustment, and that this relationship was context-dependent. Further studies that manipulate maternal condition and/or natal conditions and investigate the effects upon the lifetime reproductive success of offspring would be valuable. Overall, this study serves to highlight the complexity of sex ratio adjustment in birds and the difficulties associated with identifying sex biasing mechanisms.

## Supporting Information

Data S1 Combined raw data files.
(XLS)

Materials S1 Supporting information regarding the validation of the non-invasive technique to manipulate maternal CORT concentrations.
(DOCX)
