# Correction: Maternal Condition but Not Corticosterone Is Linked to Offspring Sex Ratio in a Passerine Bird

**DOI:** 10.1371/journal.pone.0116366

**Published:** 2014-12-23

**Authors:** The PLOS ONE Staff

The fourth author's name is spelled incorrectly. The correct name is: Aileen Adam. The correct citation is: Henderson LJ, Evans NP, Heidinger BJ, Adam A, Arnold KE (2014) Maternal Condition but Not Corticosterone Is Linked to Offspring Sex Ratio in a Passerine Bird. PLoS ONE 9(10): e110858. doi:10.1371/journal.pone.0110858
