# Supplementary material for: Maternal Condition but Not Corticosterone Is Linked to Offspring Sex Ratio in a Passerine Bird
Source: PLoS One. 2014 Oct 27;9(10):e110858. doi: 10.1371/journal.pone.0110858 (PMC4210198; doi:10.1371/journal.pone.0110858)
Supplement: Materials S1 — Supporting information regarding the validation of the non-invasive technique to manipulate maternal CORT concentrations. (DOCX) [file pone.0110858.s002.docx]

**Supplementary Material**

## *Validation of mealworm technique*

To non-invasively elevate CORT in blue tit mothers during egg laying, mothers were fed mealworms spiked with a CORT solution [[1](#_ENREF_1)]. To validate this technique blue tits were fed CORT spiked mealworms in captivity to establish whether CORT concentrations were elevated within their natural rage.

Blue tits were captured in mist nets in March 2009, in woodland on the east banks of Loch Lomond, UK (56^o^ 13′ N, 4^o^ 13′ W). Birds were then transported and realised into cages at the University of Glasgow Field Station, SCENE (*n* = 24). Birds were housed in single cages (1m x 0.5m x 0.5m) in 2 rooms (2.4m x 1.7m x 2.2m) with air, temperature and light control. The birds were kept under long-day photoperiod (12L:12D) and at 20 °C during the experiment, which was comparable to natural conditions. Birds were maintained in captivity for three days and given insectivorous mix, mealworms, waxworms and water, which were available ad libitum at all times. Birds were held in captivity for 24 hours prior to validation of mealworm technique to allow them to acclimatise to their new environment.

Experiments were conducted between 09:00 and 16:00 on the second day of captivity. In order to investigate if CORT can be non-invasively increased, pairs of birds were food deprived for half an hour and then individuals were presented with either a control or CORT spiked mealworm. Birds were paired to control for diurnal effects and disturbance, which may influence circulating CORT [[2](#_ENREF_2)]. The birds were monitored from behind a screen and their consumption of the mealworm was noted. To investigate if birds fed CORT-injected mealworms had elevated CORT compared with controls, 7 minutes after the mealworm had been consumed, birds were caught and blood sampled. Samples were collected within 3 minutes of capture to minimise effects of capture on circulating CORT concentrations [[3](#_ENREF_3),[4](#_ENREF_4)]. The timing of blood sample collection was informed by previous work employing this technique that has shown that concentrations peak at ~7 minutes after consumption [[1](#_ENREF_1)]. This procedure was repeated on a second group of birds, but on this occasion they were blood sampled ~27 minutes after consumption to determine the duration of CORT elevation.

On average birds consumed the entire mealworm within 2 minutes of presentation; only birds that consumed the entire mealworm were considered to have been successfully manipulated. 8 pairs of birds (16 individuals) were successfully manipulated and blood sampled. 4 pairs were sampled ~7 minutes after consumption and 4 pairs were sampled ~27 minutes after consumption. 8 birds could not be used; only one bird was rejected, as it did not consume the entire mealworm. The other 7 birds were not included as they were not successfully blood sampled.

Circulating CORT concentrations observed 7 minutes after mealworm consumption were significantly elevated (x13) in birds that received a CORT injected mealworm (see figure 1, *t* = -5.54, *n* = 8, *P* = 0.001). CORT concentrations did not significantly differ between birds fed CORT or control mealworms when blood sampled 27 minutes after consumption (*t* = -0.96, *n* = 8, *P* =0.41). However, birds fed a CORT injected mealworm, were on average twice as high as those fed the control mealworm (see figure 1).

**Figure 1.** Circulating CORT concentrations were significantly higher in captive blue tits fed CORT spiked rather than control mealworms ~7 minutes after consumption (*n* = 8). However, this effect was no longer evident ~27 minutes after consumption (*n* = 8). Graph depicts mean±SE

## *Natural range of circulating CORT*

In order to establish the natural range of CORT concentrations experienced by blue tits, birds were caught and blood sampled at a range of time points after capture and handling. Blue tits were captured in mist nets on two days in March 2010, on the same site as birds caught for mealworm validation. Mist nets were monitored continuously and birds were extracted within a minute of flying into the net. Once extracted from the net birds were immediately placed in bags until blood sampling.

Birds were sampled at 10 (*n* = 6), 15 (*n* = 7), 20 (*n* = 5), 25 (*n* = 5), 30 (*n* = 3) and 35 (*n* = 2) minutes after extraction from the mist net. Birds sampled 25 minutes after initial capture in a mist nest were found to have the highest mean CORT concentrations, 28.2±11.7ng/ml with a maximum of 74.6ng/ml (see figure 2). Therefore CORT concentrations elicited by feeding birds CORT injected mealworms are on average within 1 SE of circulating levels found after a bird experienced a standard restrain procedure. Furthermore manipulated CORT levels are within the maximum concentration of CORT found in birds after a standard restraint procedure.


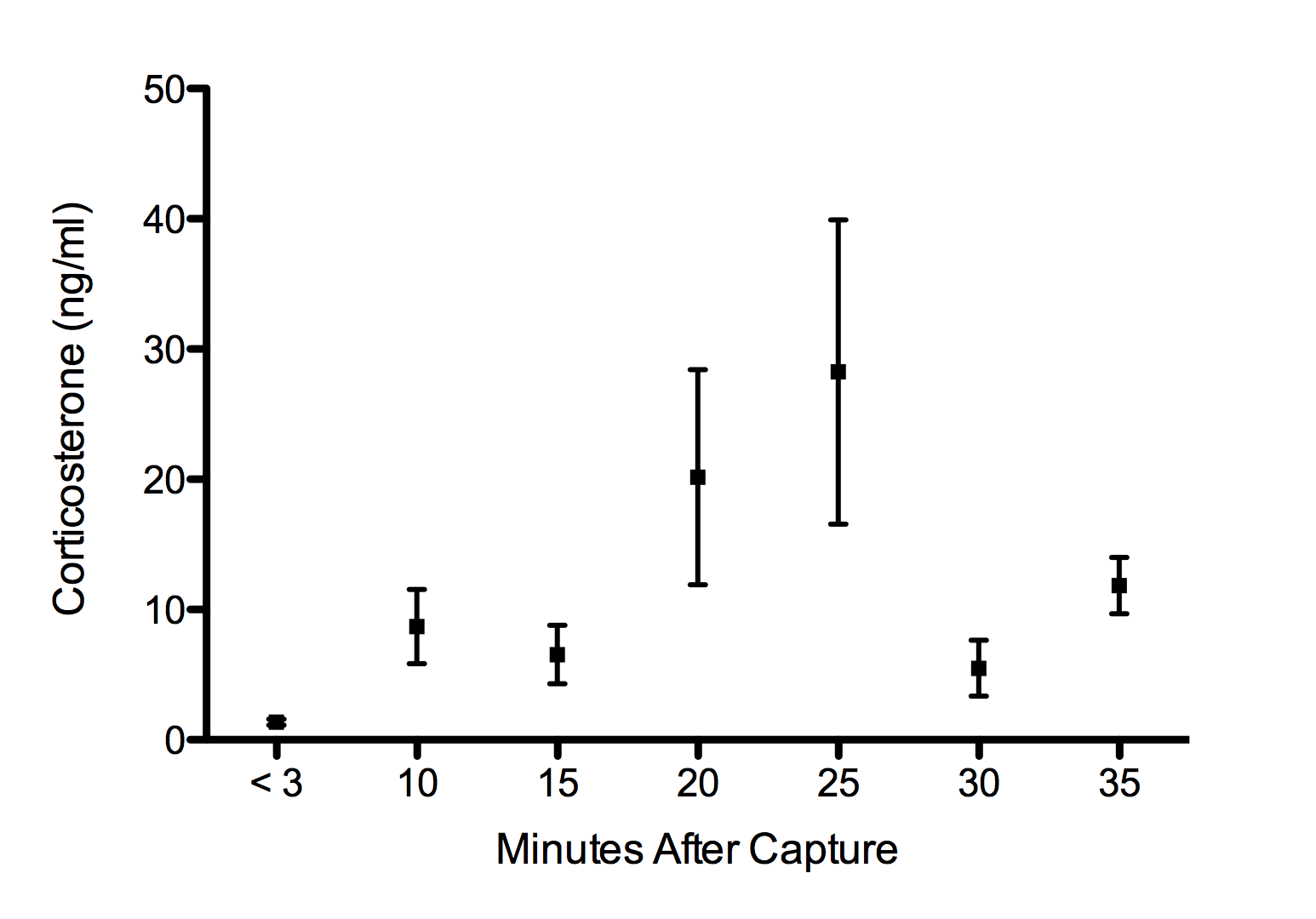


**Figure 2.** Circulating CORT concentrations measured in free-living blue tits at a range of time points after subjection to standard restraint procedure. Baseline <3 mins: *n* = 17, 10 mins: *n* = 6, 15 mins: *n* = 7, 20 mins: *n* = 5, 25 mins: *n* = 5, 30 mins: *n* = 3 and 35 mins: *n* = 2. Graph depicts mean±SE

**References**

1. Breuner CW, Greenberg AL, Wingfield JC (1998) Noninvasive corticosterone treatment rapidly increases activity in Gambel’s white-crowned sparrows (*Zonotrichia leucophrys gambelii*). General and Comparative Endocrinology 111: 386-394.

2. Romero L (2004) Physiological stress in ecology: lessons from biomedical research. Trends in Ecology & Evolution 19: 249-255.

3. Romero LM, Reed JM (2005) Collecting baseline corticosterone samples in the field: is under 3 min good enough? Comparative Biochemistry and Physiology - Part A: Molecular & Integrative Physiology 140: 73-79.

4. Wingfield JC, Smith JP, Farner DS (1982) Endocrine Responses of White-Crowned Sparrows to Environmental Stress. The Condor 84: 399-409
